# Association Between *Escherichia coli* Mastitis and Acute Laminitis in Dairy Cows

**DOI:** 10.3390/ani15121709

**Published:** 2025-06-09

**Authors:** Fabian Faustmann, Martina Baumgartner, Susanna Piechl, Birgit Fuerst-Waltl, Johann Kofler

**Affiliations:** 1 Clinical Department for Farm Animals and Food System Science, Clinical Center for Ruminant and Camelid Medicine, University of Veterinary Medicine Vienna, 1210 Vienna, Austria; fabian.faustmann@gmail.com (F.F.); susanna.piechl@vetmeduni.ac.at (S.P.); 2 Department of Sustainable Agricultural Systems, BOKU University, 1180 Vienna, Austria; birgit.fuerst-waltl@boku.ac.at

**Keywords:** mastitis, *Escherichia coli*, laminitis, lameness, survival time, cattle

## Abstract

The objective of this retrospective case-control study was to examine the relationship between *E. coli*-induced mastitis and acute laminitis in dairy cows. Correspondingly, the medical records of 93 cows diagnosed with *E. coli* mastitis between 2012 and 2023 were analysed. The cows were categorised into three groups: (1) cows with mastitis scores of 1 or 2 without signs of acute laminitis; (2) cows with a mastitis score of 3 without signs of acute laminitis; (3) cows with a mastitis score of 3 and exhibiting symptoms of acute laminitis. Out of the 93 cows with *E. coli* mastitis, 19 were assigned to group 1 (20.4%), 46 to group 2 (49.5%), and 28 to group 3 (30.1%). A total of 74 cows (79.6%) scored 3 for mastitis, but 28 cows (37.8%) were also diagnosed with acute laminitis. A higher mastitis score was significantly associated with a higher incidence of acute laminitis (*p* = 0.0004). *E. coli* mastitis showed a higher incidence in parity ≥ 3 (73.3% to 78.6%) and occurred in cows of all three groups mainly during the first third of lactation (53.6% to 75.6%). Cows in group 1 had a median survival time of 512 days, while for cows in groups 2 and 3, these values were 93 and 178 days, respectively. Regarding survival times, only a trend but no statistically significant difference (*p* = 0.221) was found among the cows in the three groups. This could possibly be explained by the fact that additional comorbidities were diagnosed in 53.6% to 65.2% of the cows from all three groups. It is concluded that cows with severe coliform mastitis have a higher risk of developing acute laminitis, and that a thorough clinical examination of the claws should always be performed to avoid overlooking acute laminitis, which accompanied the *E. coli* mastitis in approximately 38% of cases.

## Simple Summary

This retrospective study aimed to investigate the association between *E. coli*-induced mastitis and acute laminitis in dairy cows by evaluating the medical records of 93 cows diagnosed with *E. coli* mastitis. These cows were divided into three groups: (1) cows with mastitis scores of 1 or 2 without acute laminitis; (2) cows with a mastitis score of 3 without acute laminitis; (3) cows with a mastitis score of 3 and signs of acute laminitis. Nineteen cows (20.4%) were assigned to group 1, 46 (49.5%) to group 2, and 28 (30.1%) to group 3. Of the 93 cows, 74 cows (79.6%) scored 3 for *E. coli* mastitis, and 28 cows (37.8%) were also diagnosed with acute laminitis. The incidence of *E. coli* mastitis was between 73.3% and 78.6% in parity ≥3 and was observed mainly (53.6% to 75.6%) during the first 100 days of lactation. The median survival time of the cows in the three groups ranged from 93 to 512 days, without a statistically significant difference. We found that cows with severe *E. coli* mastitis had a higher probability of approximately 38% for developing acute laminitis.

## 1. Introduction

Mastitis is one of the most relevant septic diseases in dairy cows [1,2], with *Escherichia coli (E. coli)* playing a prominent role [3,4]. This facultatively pathogenic, organism is present as a coloniser of the digestive tract, and therefore also in the immediate environment of cows [1,5].

The virulence of coliform bacteria and the induction of an inflammatory response depend on their ability to multiply in the host and destroy cellular structures. During multiplication, destruction, and lysis, the Gram-negative *E. coli* bacteria release cell wall components called lipopolysaccharides (LPS). These are thermostable molecules and endotoxins [5,6]. The pathogenicity of *E. coli* in the udder is related to the release of LPS, which is considered a key molecule for the progress of this infection [4,7]. Once *E. coli* reach the teat cistern, they multiply between eight and ten-fold every two hours, releasing large amounts of LPS. Also known as microbe-associated molecular patterns (MAMPs), LPS are potent stimulators of the innate immune system and are responsible for many pathophysiological responses in the host. These include fever, changes in the number of circulating leucocytes (leukopenia, leucocytosis), complement activation, macrophage activation, and increased venous permeability leading to oedema formation, alterations in plasma levels of metabolites, minerals, acute-phase proteins and hormones [4,5,6].

Mastitis caused by *E. coli* can manifest clinically as acute [3,4], subclinical, or chronic inflammation, although the latter two forms are uncommon [8,9]. Clinical mastitis is characterised by significant changes in the milk, such as flaky, watery, or bloody secretions and changes to the udder, including redness, swelling, elevated temperature, and tenderness upon palpation [4,10]. Additionally, infection is often accompanied by a noticeable decrease in milk yield, believed to be caused by the local and systemic effects of LPS [11]. An increase in body temperature, anorexia, reduced rumen activity, dehydration, and diarrhoea may also occur [5,11,12]. In severe *E. coli* mastitis, infected mammary glands frequently exhibit extensive tissue destruction due to hypoxia [5,13]. *E. coli* infections are more common in cows with higher parity [3,12]. Severe mastitis (score 3) with concurrent symptoms of systemic disease or fatal outcomes are often observed peripartum and during early lactation, while clinical signs in mid and late lactation are usually mild to moderate [5,13,14]. During acute *E. coli* mastitis, bacteraemia can occur as a result of increased permeability of the blood–milk barrier [15,16].

Systemic Inflammatory Response Syndrome (SIRS) is a systemic inflammatory response triggered by infectious or non-infectious processes in the body [17,18]. If SIRS occurs because of an infection, the term sepsis is used to describe the complex pathological processes [18,19]. In addition to diagnosing infection through microbial identification, the following SIRS parameters have been used for calves: heart rate > 160 or <100/min, respiratory rate > 45/min, rectal body temperature < 37.0 °C or >39.5 °C, leucocytosis (>12,000/µL) or leukopenia (<4000/µL) [17,20]. However, SIRS parameters for cows have not yet been established.

Laminitis, also known as diffuse aseptic pododermatitis, is characterised by aseptic inflammation of the dermis that typically affects multiple claws in cattle [21,22,23]. The condition arises from a disruption of the microcirculation in the dermal layer of the claws, triggered by toxic and vasoactive substances such as endotoxins and histamine [23,24,25]. Endotoxins are released in significant amounts when the pH of the rumen drops for several hours per day [26], leading to the release of biogenic amines in tissues. This, in turn, causes pathological changes in the basal membrane of the epidermis of the claws [23,27,28]. Endotoxins can also increase the production and release of cytokines and metalloproteinases locally resulting in a local inflammatory response [29,30] that weakens the suspensory apparatus of the pedal bone at the lamellae of the wall segment [23,31,32].

Nutrition-related disorders, which can lead to subacute ruminal acidosis, are considered the most significant and common cause of laminitis in cattle [26,27,33]. However, septic diseases such as *E. coli* mastitis of score 3 and purulent endometritis can also result in high-grade absorption of endotoxins and histamine, potentially inducing acute laminitis [23,34].

The objectives of this retrospective study were to determine the prevalence of *E. coli* mastitis among cow patients at the University Clinic for Ruminants associated with concurrent acute laminitis. Additionally, the study aimed to examine whether breed, parity, lactation day, season of the year, and the occurrence of clinical signs consistent with septicaemia were associated with increased incidence of acute laminitis. The following three hypotheses were tested through a retrospective analysis of patient data:

**Hypothesis** **1.** *Cows with severe E. coli mastitis (score 3) are more likely to develop acute laminitis than cows with moderate (score 2) or mild mastitis (score 1).*

**Hypothesis** **2.** *Cows with E. coli mastitis and acute laminitis have a shorter survival time than cows with E. coli mastitis without acute laminitis.*

**Hypothesis** **3.** *The occurrence of acute laminitis in cattle, along with classic parameters of SIRS, can serve as a diagnostic criterion for septicaemia in cows with confirmed E. coli mastitis.*

## 2. Materials and Methods

### 2.1. Animal Data

In this retrospective case-control study, the case records of 93 cows with a positive bacteriological milk test for *E. coli* documented in the electronic ‘Tierspital-Informations-System’ (TIS) (Agfa HealthCare Orbis, Vienna, Austria) were evaluated. These cows were examined and treated for mastitis at the University Clinic for Ruminants of the University of Veterinary Medicine, Vienna, between January 2012 and December 2023. Cows that tested positive for *E. coli* in quarter milk samples and had all relevant findings from the general clinical, mammary gland, and orthopaedic (claw) examinations were included in the study. Cows bacteriologically detected for *E. coli* but without clinical symptoms at the udder and without macroscopic changes in the milk were excluded from the analyses.

All quarter milk samples were collected aseptically immediately after admission, following clinical propaedeutic guidelines [35]. Bacteriological examination was performed according to international standards [36,37]. All quarter milk samples were centrifuged at 1300× *g* for ten minutes, and 10 µL of the sediment was spread on Columbia agar (Merck KGaA, Darmstadt, Germany) supplemented with 5% sheep blood. After 24 and 48 h of incubation at 37 °C, the colonies were characterised based on their morphology (large, grey, slimy colonies with or without haemolysis), identified as *E. coli* by a positive KOH test, and lactose-positive bacterial growth in pink, dry colonies with precipitation on MacConkey agar (Oxoid). All isolates were finally confirmed as *E. coli* using MALDI-Tof MS at the Institute of Microbiology from our University.

For statistical evaluations, we collected the patient identification number, laboratory number, mastitis score (1–3), and other documented clinical findings from other organ systems. Mastitis severity was classified according to the guidelines of the International Dairy Federation [38]:

Severity score 1 (Mild): Only macroscopically detectable changes in the milk sample in the infected udder quarter (e.g., altered consistency, colour, admixture of flakes).

Severity score 2 (Moderate): Changes in milk sample and pathological findings of the infected udder quarter (swelling, altered consistency, redness, tenderness, etc.).

Severity score 3 (Severe): In addition to changes in milk secretion and pathological findings of the udder, there is also systemic disease characterised by symptoms such as fever, subnormal rectal temperature, loss of appetite, rumen stasis, etc. [38].

Subsequently, the electronically documented case records of cows diagnosed with *E. coli* mastitis were reviewed to search for clinical evidence of acute laminitis. Clinical findings that may be detectable in acute laminitis include oedematous swelling and redness of the coronary band; elevated claw temperature; pain response to palpation of the sole and wall horn using the hoof tester; bilateral lameness; stiff gait in both hindlimbs and/or all four limbs [23,39,40,41]; and a depression at the coronary band reaching from abaxial to dorsal and axial, detectable by finger palpation [42,43]. To be considered characteristic findings of acute laminitis, the signs of ‘oedematous swelling of the coronary band’, ‘elevated claw temperature’, ‘painful reaction upon palpation with the hoof tester’, and ‘depression at the coronary band’ needed to be observed in at least two or more claws of a cow. These typical signs of acute laminitis could also be accompanied by diverging horn rings on the horn wall and concave dorsal walls [22,23,39]. However, these signs of chronic laminitis were not always documented and were therefore not analysed.

Furthermore, we evaluated the following clinical parameters: rectal temperature, pulse rate, mucous membrane colour (eyelid and oral mucosa), skin elasticity, respiratory rate, rumen activity, and appetite. Rectal temperature, pulse rate, respiratory rate, and rumen activity values were recorded for five days starting from the day of milk sampling with positive detection of *E. coli*, and the median was calculated. The data published by Baumgartner and Wittek [35] served as references for these clinical parameters. Any clinical parameters deviating from these physiological limits, whether above or below, were considered pathological [35].

In addition, the ear tag number, date of birth, breed, and presence of other diseases were recorded. Using the ear tag number, the parity of the cow, date of last calving, day of lactation, and survival time after diagnosis of *E. coli* mastitis were accessed through the internet service portal of the Agrar-Markt-Austria database (eAMA; https://www.ama.at/fachliche-informationen/eama-das-internetserviceportal, accessed on 11 November 2024). To calculate the survival time of cows after a diagnosis of *E. coli* mastitis, confirmed by bacteriological examination of the quarter milk sample, the date of culling of the cows was determined by comparing the eAMA database with the individual ear tag numbers. For cows still alive at the time of the retrospective evaluation, the 11th November 2024 was set as the cut-off date for calculating the survival time after diagnosis of *E. coli* mastitis. By combining culling dates with the diagnosis dates at the clinic, we were able to determine the survival time of cows after diagnosis with *E. coli* mastitis, as well as whether the cows were still alive at that time (cumulative survival time).

Cows with *E. coli* mastitis were divided into three groups for further analysis based on their mastitis score and the presence of acute laminitis:

Group 1 included cows with *E. coli* mastitis with a clinical score of 1 or 2 [38] and without signs of acute laminitis.

Group 2 consisted of cows diagnosed with *E. coli* mastitis with a clinical score of 3 [38] displaying clinical signs at the udder, but without acute laminitis.

Group 3 included cows with an *E. coli* mastitis score of 3 [38] showing clinical signs at the udder and symptoms of acute laminitis.

Depending upon their individual prognosis, the 93 cows with *E. coli* mastitis and concurrent comorbidities either received treatment at the clinic following evidence-based guidelines [4,14,44] or did not leave the clinic alive. The results of the treatments will be published separately.

### 2.2. Statistical Analyses

Descriptive statistics for the data were generated using Microsoft^®^ Excel^®^ for Microsoft 365 MSO (Microsoft Corp., Redmond, WA, USA). Further calculations were conducted using the statistical program SAS (Version 9.4; SAS Institute, Cary, NC, USA). To investigate the increasing incidence of acute laminitis with higher severity of *E. coli* mastitis (score 1–3), a Fisher’s exact test was utilised. This calculation compared the incidence of acute laminitis with the severity of *E. coli* mastitis.

The relationship between breed (four classes: Fleckvieh, Brown Swiss, Holstein, and Crosses), stage of lactation (three classes: 1–100, 101–200, and >200 days in milk), parity (three classes: 1, 2 and ≥3), season of the year (four classes), and the number of udder quarters affected (two classes, 1 and 2) with the occurrence of *E. coli* mastitis with and without acute laminitis (groups 1, 2, and 3 as described above) were analysed separately for each effect using Fisher’s exact tests. Additionally, all effects were analysed simultaneously, using a cumulative logit model with multinomial ordered response distribution.

The association and usability of SIRS parameters as diagnostic criteria for acute laminitis and the severity of *E. coli* mastitis (treated as a continuous trait) were tested using a general linear model (GLM). Initially, all SIRS parameters and laminitis were included as fixed effects, followed by pairwise analyses of laminitis with individual SIRS parameters. Additionally, the relationship between SIRS parameters and the occurrence of *E. coli* mastitis with and without acute laminitis was investigated. The SIRS parameters considered were internal body temperature, heart rate, colour of the palpebral conjunctiva and oral mucosa, skin elasticity, respiratory rate, rumen activity, and appetite. For Fisher’s exact tests, groups 2 and 3 were combined as both had an *E. coli* mastitis severity score of 3, anticipating potential SIRS symptoms.

Furthermore, a Kaplan–Meier procedure was used to analyse the survival time following an *E. coli* mastitis diagnosis with and without acute laminitis. A relationship was considered statistically significant if the *p*-value was <0.05.

## 3. Results

A total of 93 cows with *E. coli* mastitis were included in the study. Of all cows with *E. coli* mastitis, 74 cows (79.6%) scored for 3 mastitis, and 28 cows (37.8%) developed acute laminitis. No cows with *E. coli* mastitis of scores 1 or score 2 were diagnosed with acute laminitis. Further analysis of the 93 cows showed that 19 cows (20.4%) were assigned to group 1 (mastitis score 1 or 2 without acute laminitis), 46 cows (49.5%) to group 2 (mastitis score 3 without acute laminitis), and 28 cows (30.1%) to group 3 (mastitis score 3 and acute laminitis).

Table 1 lists the respective breeds, the parity of affected cows, the days in milk and the season of the year in which *E. coli* mastitis was diagnosed, along with the infected udder quarter for the cows in the three groups. In this analysis, Fleckvieh cows were the most frequently represented, with rates ranging from 78.6% to 84.2%. It was noticeable that *E. coli* mastitis was diagnosed more frequently during parity 3 and later lactations, with rates ranging from 73.3% to 78.6%. Furthermore, *E. coli* mastitis occurred in all three groups during the first third of lactation, with rates ranging from 53.6% to 75.6%. During the last third of lactation, *E. coli* mastitis was more frequently detected in cows in group 3 (28.6%) than in cows in groups 1 (21.1%) and 2 (17.8%) (Table 1).

**Figure 1:**
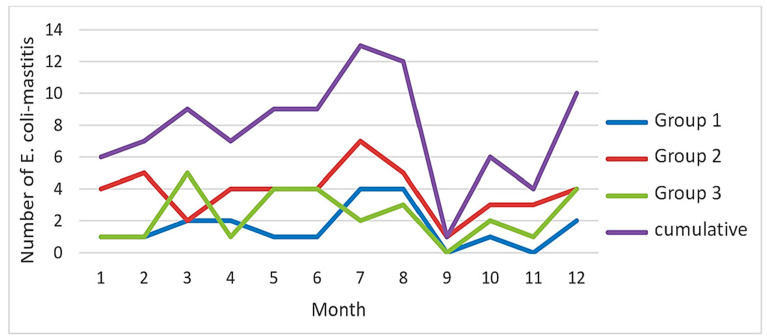
Frequency and distribution of *E. coli* mastitis in 93 cows throughout the twelve lactation months categorised by groups 1, 2, and 3 and cumulatively for all three groups combined.

Regarding seasonal variability, an uneven distribution of *E. coli* mastitis was observed throughout the year over the three groups, with a peak during the third quarter (Table 1; Figure 1). The number of *E. coli* mastitis cases showed a notable increase during the summer months of July and August (Figure 1).

In 68.4% to 75.0% of affected cows, only one udder quarter was affected, while in 25.0% to 31.6% of cases, two quarters were infected. *E. coli* mastitis was most frequently diagnosed in the right rear quarter of the udder in all three groups, with rates ranging from 28.6% to 38.3% (Table 1).

The distribution of physiological and pathological findings from the general clinical examination of cows with *E. coli* mastitis in the three groups is outlined in Table 2. In cows in groups 2 and 3, the colour of the eyelid conjunctiva, in particular, frequently showed pathological findings, with 72.1% and 78.6% of affected cows, respectively. There was also an increasing frequency of pathological findings observed regarding skin elasticity in cows in groups 2 (51.7%) and 3 (74.1%). Additionally, appetite and rumen activity were markedly impaired in all three groups (84.2% to 89.5%), with only 15.8% and 13.0% of cows in the three groups, respectively, showing physiological values for these two para-meters [35]. However, cows in group 1 also displayed a high percentage of pathological findings in the eight SIRS parameters ranging from 26.3% (oral mucosa) to 89.5% (rumen activity). These findings are not typically expected in cases of *E. coli* mastitis with a score of 1 or 2 (Table 2).

The frequency and types of findings characteristic of acute laminitis observed during the claw examination of 28 cows suffering from *E. coli* mastitis and acute laminitis are listed in Table 3. In all cows with acute laminitis, a depression at the coronary band in two or more claws was detectable by finger palpation (100.0%). Positive pain palpation of the claws using the hoof tester was only observed in 63.3% of the cows, and an elevated claw temperature was only found in 60.7% of the cows with acute laminitis. All 28 cows exhibited a stiff gait and lameness (Table 3).

*E. coli* mastitis was present in all cows upon admission to the clinic. Among the 28 cows with *E. coli* mastitis, nine (32.1%) showed signs of acute laminitis during initial examination at the clinic. The remaining 19 cows (67.9%) developed signs of acute laminitis between 24 and 96 h after admission.

In addition to *E. coli* mastitis and acute laminitis, other diseases were also diagnosed in these cows. In group 1, 10 out of 19 cows (57.9%); in group 2, 30 out of 46 cows (65.2%); and in group 3, 15 out of 28 cows (53.6%) exhibited additional organ diseases (Table 4).

Cows in group 1 demonstrated the highest survival time with a median of 512 days (mean: 743.6; SD: 643.3). In comparison, cows in group 2 had a median survival time of 93 days (mean: 425.3; SD: 503.1), while cows in group 3 survived for 178 days (mean: 444.3; SD: 581.1).

At the cut-off date, a total of seven cows were still alive (and thus censored). Among them, two (10.5%) were in group 1, three (6.5%) were in group 2, and two cows (7.1%) were in group 3. The two cows in group 1 had a median survival time of 1851.5 days (mean: 1851.5 days; SD: 44.5), the three cows in group 2 had a median of 1312 days (mean: 1235.6 days; SD: 286.2), and the two cows in group 3 had a median of 482.5 days (mean: 482.5 days; SD: 214.2).

**Figure 2:**
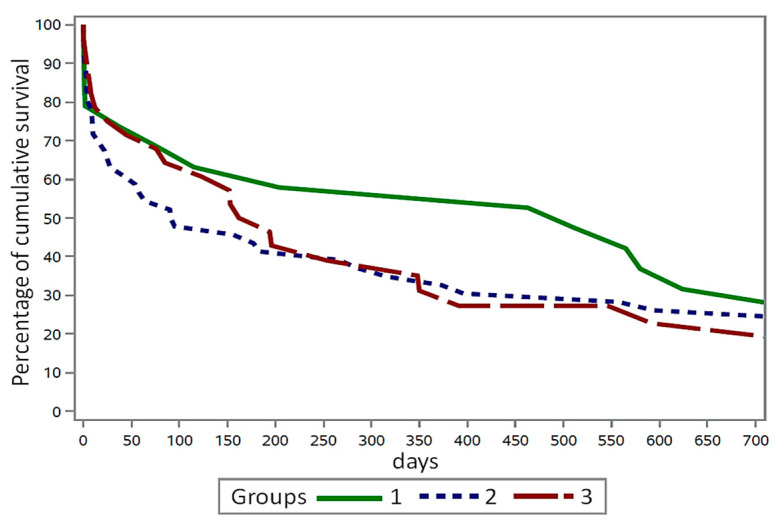
Smoothed Kaplan–Meier survival curve of 93 cows from the three groups displaying the cumulative survival rate up to 700 days after positive sampling of *E. coli*, indicating detection of *E. coli* mastitis.

The cumulative survival rate of cows in the three groups after the diagnosis of *E. coli* mastitis is depicted in Figure 2. The graph illustrates that cows in group 2 experienced a mortality rate of 51.8% up to day 100 following the diagnosis of *E. coli* mastitis, in comparison to cows in group 1 (35.2%) and group 3 (38.1%). By day 200 after *E. coli* diagnosis, 49.2% and 48.1% of the cows in groups 2 and 3 had already expired, respectively.

Table 5 displays the number of cows that were euthanised or died during their hospitalisation due to the worsening of the disease(s) despite treatment. This affected 21 out of 93 cows (22.9%), while the remaining cows were returned to their owners.

### Results of Statistical Analyses

Testing for a potential increase in the incidence of acute laminitis with higher mastitis severity (score 1–3) using Fisher’s exact test revealed a table probability of *p* = 0.0004. This indicated a significantly higher occurrence of acute laminitis with a higher mastitis score (score 3). The differences in the survival rates of the cows in the three groups (*E. coli* mastitis with and without acute laminitis) are depicted in Figure 2. However, only a trend and not a statistically significant difference was observed in survival time between the cows in the three groups (*p* = 0.221; log-rank test of the Lifetest procedure).

Analysis of variance using the GLM procedure indicated that, while keeping all other variables constant, the presence of acute laminitis was significantly associated with the severity of mastitis (*p* = 0.012). However, the other independent variables, such as internal body temperature (*p* = 0.773), heart rate (*p* = 0.709), colour of the palpebral conjunctiva (*p* = 0.128), colour of the oral mucosa (*p* = 0.215), skin elasticity (*p* = 0.244), respiratory rate (*p* = 0.183), rumen activity (*p* = 0.418), and appetite (*p* = 0.702) showed no significant association with the occurrence of acute laminitis. This was the case even when each variable was considered individually in conjunction with laminitis as an effect in the model.

Both test regimens, the Fisher’s exact test for each trait separately and the cumulative logit model (Table 1) revealed no statistically significant association for the variables breed (*p* = 0.932 and *p* = 0.656), parity (*p* = 0.952 and *p* = 0.697), lactation day (*p* = 0.370 and *p* = 0.419), season of year (*p* = 0.397 and *p* = 0.139), and udder quarter (*p* = 0.870 and *p* = 0.630), respectively, in relation to the occurrence of *E. coli* mastitis with and without acute laminitis.

The statistical analysis of the presence of SIRS parameters in cows suffering from *E. coli* mastitis with a score of 3 (with or without acute laminitis, groups 2 and 3), compared to cows in group 1 suffering from *E. coli* mastitis with a score of 1 or 2 revealed significant differences only for the colour of eyelid conjunctiva (*p* = 0.029) and a trend in skin elasticity (*p* = 0.066) (Table 2).

## 4. Discussion

Published studies on the potential impact of mastitis on the development and incidence rate of claw disorders are scarce. Only Motamedi et al. [45] and Griffiths et al. [46] described a relationship between mastitis incidence and the occurrence of claw horn lesions. Other authors mentioned that ‘peracute’ *E. coli* mastitis can lead to acute or subacute laminitis [23,34], but they did not provide further information on the prevalence and prognosis of these concurring conditions. Motamedi et al. [45] assessed mastitis diagnoses using the International Dairy Federation definitions with three severity levels [38]. They also analysed data from biannual hoof trimming procedures (the second trimming occurred three months after the mastitis diagnosis) and monthly gait assessments of 800 cows in a dairy herd over a nine-months period. A total of 543 cows were diagnosed with and treated for mastitis during this period. The control group comprised the same number of cows that had not been diagnosed with mastitis during the previous three months. However, the authors did not provide any information about the bacteriological findings of their mastitis cases [45]. The incidence of white line lesions and sole ulcers was twice as high in the mastitis group (2.9%) as in the control group (1.3%), although this difference was not statistically significant. However, the incidence of lameness was significantly higher in cows in the mastitis group (8.1%) compared to the control group (5.2%) [45]. The authors concluded that scores 2 and 3 of mastitis have clinical relevance in relation to the increased incidence of claw horn lesions. In the classification of mastitis, score 2 mastitis was predominantly reported in this study (90.8%), whereas only 1.5% of cows had score 3 and 7.7% had score 1 mastitis [45].

In this retrospective study of 93 cows, only cows with *E. coli* mastitis were assessed. Out of the total, only 20.4% had scores 1 and 2 mastitis, while 79.6% had score 3. This skewed distribution may be attributed to the selective patient material used. Cows with mild or moderate mastitis are typically not referred to the clinic as they can be easily treated in a practical setting. A study from Germany, which involved 58 herds, found that 27.6% of clinical mastitis cases were diagnosed as score 3. The incidences of score 2 (38.2%) and score 1 mastitis (34.2%) were nearly equal in comparison [2]. In cases of score 3 mastitis, coliform bacteria were the most commonly detected at 52.2% [2]. In addition to *E. coli* a wide range of Gram-negative bacteria, including *Klebsiella* sp., *Enterobacter* sp., *Serratia* sp., and *Pseudomonas* sp., also possess LPS as a virulence factor being a potent activator of the immune system and may therefore also be associated with laminitis [2,12].

When considering the prevalence of *E. coli* mastitis in relation to the parity of the cows, 78.6% of mastitis cases were diagnosed during lactations three and higher. In contrast, Motamedi et al. [45] found mastitis with unknown bacteriological agents predominantly in the first three lactations (60.4%). Other authors reported a more frequent prevalence of *E. coli* mastitis or severe forms of *E. coli* mastitis in cows in higher parity [5,12,13,44]. Other studies reported a higher prevalence of *E. coli* mastitis or severe forms of *E. coli* mastitis in cows of higher parity. They explain this by pointing to a better immune competence in primiparous cows [5,12,13], as well as an increasing risk of predisposing factors (such as ketosis, lameness, and the impact of the milking machine) in multiparous cows [2]. As frequently reported by others [5,12,44], *E. coli* mastitis was diagnosed during the first third of lactation in the cows of all three groups in the present study, with rates ranging from 53.6% to 75.6%. In the last third of lactation, it was notable that cows in group 3 were more frequently infected (28.6%) than cows in groups 1 (21.1%) and 2 (17.8%), although this difference was not statistically significant.

The analysis of the number of *E. coli* mastitis cases in 93 cows over the course of the year showed a significantly higher incidence in the summer months of July and August. This result is consistent with reports from other authors who have also noted a higher risk of udder infections with coliform bacteria during the summer months. Heat stress has been cited as a possible explanation [12,47]. In cases of mastitis caused by environmentally associated pathogens, including *E. coli*, only one quarter of the udder is affected in most cases [8,45]. This was also confirmed in the present study for the cows in all three groups.

In our study, 80.6% of the 93 cows evaluated belonged to the Simmental breed, while the remaining cows were of other breeds. This percentage closely mirrors the distribution of dairy cattle breeds in Austria [48]. Genetic factors may play a role in susceptibility to mastitis, with high yielding breeds appearing to be more vulnerable [49]. However, the immune response to mastitis is genetically highly conserved across breeds, with environmental factors, the host, or the pathogens involved having a greater impact on mastitis outcomes than breed [4,5,49].

In clinical *E. coli* mastitis, along with changes in milk secretion and evident inflammatory signs in the affected udder quarter, a marked decrease in milk yield, an increase in internal body temperature, anorexia, reduced rumen activity, dehydration, diarrhoea, and other general clinical symptoms are often observed [4,10,12]. Some of these clinical symptoms are the result of a systemic inflammatory response [17,18,19,20] and can be attributed to the local and systemic effects of LPS from the cell wall of Gram-negative *E. coli* bacteria [4,11,16]. Pathological clinical findings related to the presence of SIRS parameters [17,18,19,20] were observed in cows in all three groups. Specifically, changes in the colour of the eyelid conjunctiva were noted in 72.1% and 78.6% of cows, respectively, followed by alterations in skin elasticity at 51.7% and 74.1%, respectively. Appetite and rumen activity were clearly impaired in 84.2% to 89.5% of cows in all three groups. However, when comparing the SIRS parameters in cows suffering from *E. coli* mastitis with a score of 3 (with or without acute laminitis, groups 2 and 3) to cows in group 1 suffering from *E. coli* mastitis with a score of 1 or 2, a statistically significant association was only found for the colour of the eyelid conjunctiva (*p* = 0.029), with a trend observed in skin elasticity. Similar results were reported in cows with acute *E. coli* mastitis, where no significant differences were found between survivors and non-survivors in terms of internal body temperature, heart rate, and respiratory rate [13].

Statistical analysis of the data from the 93 cows demonstrated a significantly higher risk of developing acute laminitis in cows with a score of 3 mastitis. Out of the 93 cows, all 28 suffering from acute laminitis were diagnosed with *E. coli* mastitis with a score of 3. No cows with *E. coli* mastitis with scores of 1 and 2 developed acute laminitis. This result likely indicates a direct causal relationship between the presence of *E. coli* mastitis score 3 and the slightly delayed onset of acute laminitis. Some of the other organ diseases listed in Table 4 also have the potential to trigger acute laminitis. However, no acute laminitis was diagnosed in the cows in groups 1 and 2, even though 57.9% and 65.2% of the cows in these two groups, respectively, had additional and partly severe comorbidities.

In the present study, 73.3% to 78.6% of mastitis cases were diagnosed in parity 3 and higher. The 78.6% percentage refers to the 28 cows with *E. coli* mastitis and concurrent acute laminitis. Why not all cows (only 37.8%) with *E. coli* mastitis with a score of 3 also developed acute laminitis remains speculative. The literature cites individual cow-associated factors such as different susceptibilities due to impaired immune defence, differences in the virulence of the causative *E. coli* strains, individual factors such as age, lactation stage of the cows [3,5,44], and low milk protein content of less than 3.4% at the herd level [2].

In studies on score 3 mastitis [10,15] and in numerous studies on *E. coli* mastitis [4,8,13,44], there is no evidence reported of acute laminitis in the affected cows. In the study by Oliveira et al. [10], *E. coli* was most frequently detected in 741 cows with clinical mastitis (22.5%), followed by other pathogens. From this, one could conclude that when diagnosing severe cases of *E. coli* mastitis, potentially concomitant acute laminitis is clearly not receiving enough attention. This may be because the entire patient is not being interpreted, and the operators may not yet be aware of the high probability of simultaneous concurrence of *E. coli* mastitis of score 3 and acute laminitis.

A possible explanation for the significantly higher incidence of acute laminitis in cows with an *E. coli* mastitis score of 3, in older cows with parity ≥ 3 (78.6%), compared to only 7.1% in first lactation and 14.3% in second lactation, could be that cows in higher parity had previously experienced subclinical or subacute laminitis [26,27,33]. These conditions may have developed because of subacute ruminal acidosis, which could have already caused damage to the suspensory apparatus of the pedal bone [23,29,31]. This pre-existing damage to the lamellae of the claw wall dermis could promote the development of acute laminitis due to a high-grade and persistent accumulation of endotoxins in the dermal capillaries over several days, as seen in *E. coli* mastitis score 3 [3,6].

It is well documented in the literature that heifers and cows in their first lactation have a low prevalence and severity of claw lesions, unlike cows in later lactations [33,48,50]. The presence of other organ diseases, especially during the peripartum period, can also have a significant impact on susceptibility to *E. coli* mastitis [5,6]. In the three groups studied, 53.6% to 65.2% of cows had one or more, sometimes serious, comorbidities in addition to *E. coli* mastitis, as also reported by other authors [5,13,14].

In the study by Oliveira et al. [10], the culling rate from the herd due to clinical mastitis within 90 days of diagnosis was 18.1%; of these, 35.0% had diagnosis of *E. coli* mastitis. In the current study, cows in group 1 had the longest survival time after *E. coli* mastitis diagnosis, with a median of 512 days. This was followed by cows in group 3 with a median of 178 days, and cows in group 2 with the shortest survival time, at a median of 93 days. These results align with Oliveira et al.’s [10] findings on survival time for cows in group 2 with an *E. coli* mastitis score of 3 but no acute laminitis. However, statistical analysis showed no significant difference in survival time between the three groups. Other comorbidities present simultaneously also impact the survival time of cows diagnosed with *E. coli* mastitis, with or without acute laminitis. As mentioned earlier, 53.6% to 65.2% of cows in the three groups had one or more comorbidities in addition to *E. coli* mastitis, some of which were serious, particularly in cows in group 2. These unfavourable conditions were a decisive factor in 21 of 93 cows (22.9%) that were euthanised or died during hospitalisation.

There is no information in the literature regarding the duration of the prepatent phase until the first characteristic signs of acute laminitis are detectable after *E. coli* mastitis with a score of 3. In contrast, there are numerous studies that have investigated the duration of the period from experimental induction by oligofructose administration to the first onset of acute laminitis symptoms in cattle. For example, Thoefner et al. [25] observed the onset of lameness in 66% of Holstein heifers 39 h after the induction of ruminal acidosis. In further studies, lameness and permanent weight shifting were observed in 100% of cattle, on average, 72 (60–120) hours after the experimental induction of ruminal acidosis [30,32,40]. However, only 43% [32], up to 83% [25], and up to 93% of the animals [40] showed a painful reaction to claw palpation using the hoof tester in these experimental studies. The first positive pain responses using the hoof tester were detected 24 to 72 h after the induction of ruminal acidosis [25,32,40]. Using thermography, a sensitivity of 96%, a specificity of 63%, and an accuracy of 75% were reported for the detection of acute laminitis, with the hoof tester palpation as the gold standard [40].

Evaluation of several feeding studies that aimed to induce subacute ruminal acidosis by administering large amounts of concentrate in the ration showed that the first signs of acute laminitis, such as lameness and continuous weight shifting, were observed approximately 39–120 h after the start of this ration [41]. The data from the literature are consistent with clinical observations in 19 cows (67.9%), with *E. coli* mastitis score 3 and acute laminitis, where signs of acute laminitis were detectable 24 to 96 h after admission to the clinic.

None of the studies mentioned cited depression at the coronary band as a characteristic clinical sign for acute laminitis [25,30,32,40]. However, this easily palpable finding has long been used for the clinical diagnosis of acute laminitis in horses [42] and has been considered the gold standard for clinical detection of acute laminitis in cattle at our clinic for many years [43]. The depression at the coronary band in several claws was palpable in all 28 cows with acute laminitis. In comparison, other clinical findings suggestive of acute laminitis, such as a positive pain response to palpation using the hoof tester, were present in only 63.3%, elevated claw temperature due to aseptic inflammation in acute laminitis in 60.7%, and oedematous swelling and redness of the peripheral ligament only in 53.6% of cows with acute laminitis. A stiff gait in both hind limbs and/or all four limbs, as well as lameness, was observed in all cows with acute laminitis. However, these changes in gait do not necessarily indicate acute laminitis but can also be observed in many disorders present on the claws of both hindlimbs, such as sole ulcers, white line abscesses, acute digital dermatitis, etc. [46,51].

Griffiths et al. [46] attributed the occurrence of sole ulcers in cows that developed mastitis within 30 days of calving to the presence of systemic inflammation during early lactation. This inflammation damages the suspensory apparatus of the pedal bone. Other authors also reported associations between the occurrence of mastitis during the service period and the occurrence of laminitis and lameness, although they did not provide further details [23,34]. Both endotoxins and histamine can enter the bloodstream in cases of *E. coli* mastitis with a score of 3, and thus also reach the small dermal capillaries of the claws. This leads to intravascular blood coagulation and the opening of arteriovenous shunts, so that a large portion of the blood is not further transported to the dermis [21,23,24,27]. This disruption of microcirculation leads to an insufficient supply of oxygen and nutrients to the dermis. The reduced perfusion leads to the loss of the strong connection between the dermis and the epidermis [23,28,31]. The mechanical stress on this weakened connection due to the animal’s body weight causes the dermal–epidermal junction of the suspensory apparatus to detach, resulting in sinking of the pedal bone within the horn capsule [25,31,32]. These aseptic inflammatory processes in the dermis and sinking of the pedal bone cause the appearance of the characteristic clinical symptoms of acute laminitis described above [23,40,41]. The sinking of the pedal bone within the horn capsule in the case of acute laminitis also causes the tissue depression around the coronary band, which can be palpated using the finger [42,43]. Sinking of the pedal bone leads to further damage to the dermis, especially on the sole, as it is crushed between the flexor tubercle of the pedal bone and the sole horn. This results in additional localised bleeding and bruising of the dermis of the sole directly under the flexor tubercle, which can then result in externally detectable sole haemorrhages, double soles, white line lesions, and sole ulcers within six to eight weeks [22,23,28,39].

Hypothesis 1 was confirmed by the analysis of data from 93 cows, which suggested that those with severe *E. coli* mastitis (score 3) are more likely to develop acute laminitis compared to cows with moderate (score 2) or mild mastitis (score 1). None of the cows with *E. coli* mastitis scored as 2 or 1 showed signs of acute laminitis. However, of the 74 cows with *E. coli* mastitis of score 3, only 37.8% developed acute laminitis.

Hypothesis 2, which stated that cows with *E. coli* mastitis and acute laminitis have a shorter survival time than cows with *E. coli* mastitis but no acute laminitis, could only be partially confirmed by analysis of the available data. Regarding the survival rate of the cows, only a trend but no statistically significant difference could be observed between the cows of the three groups. Similar results to those for the cows in group 2 were reported by Oliveira et al. [10]. In their study, 18.1% of the cows with clinical mastitis were culled from the herd within 90 days of diagnosis. In contrast, in the present study, no differences in the culling rate were detectable for cows in groups 2 and 3 up to the 200th day following the diagnosis of *E. coli* mastitis (49.2% versus 48.1%). Furthermore, it must also be considered that some of the additional organ diseases diagnosed may have comparable impact on the survival time as the *E. coli* mastitis itself.

Hypothesis 3, which suggested that acute laminitis in cattle, in addition to classic parameters of SIRS, could be used as a diagnostic criterion for septicaemia in cows with diagnosed *E. coli* mastitis, could not be confirmed. However, no significant differences were found between the cows in group 1 compared to the cows in groups 2 and 3, the latter suffering from a severe form of *E. coli* mastitis, in terms of the SIRS parameters considered with the exception of the colour of the eyelid conjunctiva. Cows in group 1 suffering from score 1 or score 2 *E. coli* mastitis also exhibited abnormal clinical values in the SIRS parameters. However, these clinical abnormalities could potentially be linked to other concurrent diseases listed in Table 4, rather than being caused by score 1 and score 2 *E. coli* mastitis.

This retrospective case-control study also has some limitations. The sample sizes in the subgroups with 19, 28, and 46 cows are too small to draw general conclusions. The association between *E. coli* mastitis and laminitis was intentionally not analysed using logistic regression to account for additional effects, as none of the cows with mastitis scores 1 or 2 were diagnosed with acute laminitis. Despite this, descriptive statistics were used, and Fisher’s exact test was applied under the assumption that the lack of laminitis cases did not indicate structural zeros [52]. Furthermore, this field study with hospital patients could not account for all possible influencing factors, such as the duration of the udder inflammation prior to admission to the clinic, and the type and duration of treatment provided by the referring veterinarian. The available data from the 93 clinical patients were not suitable for testing Hypothesis 3, as the cows in all three groups suffered from concurrent, sometimes serious, organ diseases in 53.6% to 65.2% of cases. Particularly in group 1 cows with *E. coli* mastitis of score 1 and 2, as many as 57.9% suffered from comorbidities. A prospective or experimental study would likely be appropriate to properly test Hypothesis 3. By conducting a future prospective multicentre study in several large commercial dairy farms, the association between coliform mastitis and acute laminitis, as well as the underlying pathophysiological mechanisms, could be addressed with a much larger number of cows. This approach would help to avoid bias and improve the rigor of the results.

## 5. Conclusions

In addition to the existing, albeit limited, literature on the relationship between mastitis and acute laminitis, the findings of this study are anticipated to encourage veterinarians to conduct a comprehensive examination of a patient’s claws as standard practice when diagnosing a cow with *E. coli* mastitis score 3. The study, which involved 93 cows, revealed that those with a score of 3 for *E. coli* mastitis have a 38% chance of developing acute laminitis within a few days. In cows suffering from multiple health problems, a thorough assessment of the prognosis is essential to determine if therapy is appropriate. If so, consistent treatment of acute laminitis is just as important as consistent treatment of the underlying *E. coli* mastitis and the other concurrent disorders.

## Figures and Tables

**Table 1:** Distribution of animal-specific variables among the 93 cows in groups 1, 2, and 3 with *E. coli* mastitis (^1^: ratio of case numbers within the respective group; ^2^: Fleckvieh x Holstein crossbreeds, Fleckvieh x Red Holstein crossbreeds; ^3^: DIM = days in milk—lactation day; Q: quarter of the year); *p*-values for Fisher’s exact tests (p_Fish_) and a cumulative logit model including all effects (p_cum_) for values 1, 2, and 3 representing groups 1, 2, and 3.

	Group 1		Group 2		Group 3		p_Fish_	p_cum_
	*n*	% ^1^	*n*	% ^1^	*n*	% ^1^		
Number	19	20.4	46	49.5	28	30.1		
Breed							0.932	0.656
Fleckvieh (Simmental)	16	84.2	37	80.4	22	78.6		
Holstein	3	15.8	5	10.9	3	10.7		
Brown Swiss	0	0.0	1	2.2	1	3.6		
Others ^2^	0	0.0	3	6.5	2	7.1		
Parity	19		46		28		0.952	0.697
1	2	10.5	3	6.7	2	7.1		
2	3	15.8	9	20.0	4	14.3		
≥3	14	73.7	33	73.3	22	78.6		
Lactation days	19		46		28		0.370	0.419
DIM ^3^ 1–100	13	68.4	34	75.6	15	53.6		
DIM ^3^ 101–200	2	10.5	3	6.7	5	17.9		
DIM ^3^ > 200	4	21.1	8	17.8	8	28.6		
Season (quarter) of year	19		46		28		0.397	0.139
Q1	4	21.1	11	23.9	7	25.0		
Q2	5	26.3	12	26.1	9	32.1		
Q3	9	47.4	13	28.3	5	17.9		
Q4	1	5.3	10	21.7	7	25.0		
Infected quarter							0.870	0.630
One	13	68.4	32	69.6	21	75.0		
Two	6	31.6	14	30.4	7	25.0		
Right front	3	12.0	10	16.7	7	20.0		
Right rear	8	32.0	23	38.3	10	28.6		
Left front	7	28.0	10	16.7	8	22.9		
Left rear	7	28.0	17	28.3	10	28.6		

**Table 2:** Distribution of the physiological and pathological findings of the clinical examination in 93 cows suffering from *E. coli* mastitis; ^1^: ratio of the number of cases within the respective group; ^2^: physiological body temperature 38.3–38.8 °C; ^3^: physiological pulse rate 60–80/min; ^4^: colour of eyelid conjunctiva and oral mucosa physiologically “pale pink”; ^5^: skin elasticity physiologically “preserved” (skin fold disappears within 1–2 s]; ^6^: physiological respiratory rate 10–30 breaths/min; ^7^: rumen activity physiologically 5/5 min; ^8^: physiological “feed intake not disturbed”. The published data by Baumgartner and Wittek [35] served as a reference for these clinical findings. *p*-values for Fisher’s exact tests (p_Fish_) are shown for the comparisons of group 1 vs. groups 2 + 3.

	Group 1		Group 2		Group 3		p_Fish_
	*n*	% ^1^	*n*	% ^1^	*n*	% ^1^	
Number (*n*)	19		46		28		
Rectal temperature ^2^	19		46		28		0.802
physiological	9	47.4	24	51.1	14	50.0	
pathological	10	52.6	22	48.9	14	50.0	
Puls rate ^3^	19		46		28		0.611
physiological	12	63.2	26	56.5	15	53.6	
pathological	7	36.8	20	43.5	13	46.4	
Conjunctiva ^4^	19		43		28		0.029
physiological	10	52.6	12	27.9	6	21.4	
pathological	9	47.4	31	72.1	22	78.6	
Oral mucosa ^4^	19		43		28		0.520
physiological	14	73.7	38	88.4	20	71.4	
pathological	5	26.3	5	11.6	8	28.6	
Skin elasticity ^5^	18		42		27		0.066
physiological	11	61.1	18	42.9	7	25.9	
pathological	7	38.9	24	57.1	20	74.1	
Respiratory rate ^6^	26		46		28		0.495
physiological	15	57.7	19	41.3	16	57.1	
pathological	11	42.3	27	58.7	12	42.9	
Rumen activity ^7^	19		46		28		1.000
physiological	2	10.5	6	13.0	3	10.7	
pathological	17	89.5	40	87.0	25	89.3	
Feed intake ^8^	19		44		28		0.709
physiological	3	15.8	6	13.6	3	10.7	
pathological	16	84.2	38	86.4	25	89.3	

**Table 3:** Clinical findings on the claws in 28 cows with acute laminitis.

Findings	Number of Cows	%
Palpable depression at the coronary band on two or more claws	28	100.0
Stiff gait on both hindlimbs/on all four limbs	28	100.0
Lameness on several limbs	28	100.0
Positive pain palpation of the claws using a hoof tester on two or more claws	18	63.3
Elevated temperature on two or more claws	17	60.7
Oedematous swelling and redness of the coronary band on two or more claws	15	53.6

**Table 4:** Overview of the type and prevalence of other organ diseases in the 93 cows suffering from *E. coli* mastitis with or without acute laminitis (groups 1–3). Several cows were diagnosed with more than one of these additional diseases.

	Group 1		Group 2		Group 3	
	*n*	% ^1^	*n*	% ^1^	*n*	% ^1^
Total number of other organ diseases	11	57.9	30	65.2	15	53.6
Gastrointestinal diseases	4	21.0	15	32.6	4	14.3
*Abomasal displacement*	4	21.0	7	15.2	3	10.7
*Haemorrhagic bowel syndrome*	0	0	4	8.7	0	0
*Others*	0	0	3	6.5	1	3.6
Claw disorders (*Claw horn lesions*, *infectious claw diseases*, *deep digital sepsis*)	3	15.8	4	8.7	2	7.1
Teat lesions	2	10.5	3	6.5	2	7.1
Metabolic diseases *Subclinical ketosis*, *calcium deficiency*	1	5.3	3	6.5	3	10.7
Ovarian disease	1	5.3	0	0	0	0
Endometritis	0	0	6	13.0	4	14.3

**Table 5:** Number of cows among the three groups that were either euthanised or died during hospitalisation, as well as those that were discharged back to the farmers.

	Group 1		Group 2		Group 3	
	*n*	% ^1^	*n*	% ^1^	*n*	% ^1^
Euthanised/died during hospitalisation	4	21.1	9/3	20.0/6.7	5	17.8
Discharged from the clinic	15	78.9	33	73.3	23	82.1
Still alive on deadline (11 November 2024)	2	10.5	3	6.7	2	7.1

## Data Availability

The data used in the current study are not publicly available due to privacy restrictions of the patient data and the patient owners.
